# Installation of a stationary high desk in the workplace: effect of a 6-week intervention on physical activity

**DOI:** 10.1186/s12889-015-1724-3

**Published:** 2015-04-12

**Authors:** Motohiko Miyachi, Satoshi Kurita, Julien Tripette, Ryo Takahara, Yoshiko Yagi, Haruka Murakami

**Affiliations:** Department of Health Promotion and Exercise, National Institute of Health and Nutrition, 1-23-1 Toyama, Shinjuku, Tokyo 162-8636 Japan; ITOKI Inc, Chuo, Tokyo Japan

**Keywords:** Standing work, Occupational activity, Sedentary, Workplace

## Abstract

**Background:**

Extended sitting time at work is viewed as a crucial public health issue. Encouraging workers to stand during their office hours via the installation of standing desks maybe one effective option to combat this. Here, we investigate whether the installation of high desks in the workplace can induce positive changes in the amount of physical activity (PA) and thereby lead to subsequent improvements in anthropometric parameters.

**Methods:**

Thirty-two white-collar workers (22 men and 10 women, mean age 44.2) were randomly divided into two groups. A randomised crossover trial was performed for 13 weeks. During the experimental period, subjects completed their office work in a standing position using stationary high desks (standing work, SW) for 10 hours per week or more (SW period). The subjects were asked to maintain their normal sitting working habits during the control period (CONT period). The primary outcome was PA, which was assessed objectively using a triaxial accelerometer during weekdays and weekends. The secondary outcomes were anthropometric measurements. For each group and each parameter, the mean values during each period were recorded and were compared by paired *t* test.

**Results:**

The daily total PA (10.2  ±  2.4 vs. 9.7  ±  2.3 METs · h/day, *P* = 0.043), MVPA (4.2  ±  2.2 vs. 3.7  ±  1.8 METs · h/day, *P* = 0.025), time spent in moderate PA (58.2 ± 20.7 vs. 53.4 ± 17.0 min/day, *P* = 0.019) and time spent in MVPA (62.8 ± 25.1 vs. 57.0 ± 20.3 min/day, P = 0.019) were significantly higher during the SW period compared to the CONT period. A weekdays verses weekends subanalysis revealed that these parameters were significantly higher during the SW period compared to the CONT period during weekdays only. No significant differences were noted before and after SW periods for most of the anthropometric measures, except waist circumference (83.7  ±  7.9 vs. 83.0  ±  7.9 cm, respectively, *P* = 0.007).

**Conclusions:**

Standing work, via the installation of high desks, significantly increases moderate to vigorous physical activity, especially on weekdays.

**Trial registration:**

UMIN-CRT, UMIN000016731, 7th March 2015.

## Background

Prolonged sitting time at work is viewed as a crucial public health issue. Several studies indicated that sedentary behaviour (including sitting) is associated with higher rates of mortality and elevated incidences of cardiovascular diseases, diabetes and depression [1-5]. A recent study by Bauman *et al.* [6] reported that Japanese young adults have the longest sitting time out of 20 different developed countries. This may be a consequence of the long working times among office workers. Therefore, effective countermeasures to decrease the sitting time at work may be a powerful health promotion strategy in Japan, as well as in all countries with a large number of white-collar employees.

Encouraging workers to stand during their office hours, via the installation of standing desks (standing work, SW) or sit–stand workstations, maybe one viable approach to increasing physical activity (PA) at work. Previous studies have shown high level of acceptability [7,8] as well as significant reductions in sitting time among office workers [7,9-11]. Moreover, SW has been shown to be associated with various positive health effects, such as improvement in employees’ mood and a reduction in work-related musculoskeletal discomfort, such as neck and back pain [10,12]. In addition, Thorpe *et al.* [13] described an attenuation of the postprandial glycaemic response in employees undergoing a SW intervention. If we consider a long-term intervention, SW could be viewed as a powerful prevention strategy against the development of chronic metabolic disorders in office workers [13].

Despite these data, the literature on SW is still limited and somewhat contrasted [14]. In particular, it is unclear whether SW interventions can have a significant effect on the overall level of PA. Most studies used sit-stand workstations, which are height adjustable desks [7,10-13,15]. These studies described a significant but very slight increase in the overall PA (e.g. stepping time = + 6 min per day after one week [7]) and did not differentiate between light-intensity PA (LPA) and moderate-to-vigorous PA (MVPA). In comparison, Gilson *et al.* [9] used “hot” SW desks shared between employees and installed away from their usual desks. Such a setting might be able to promote movements between the sitting and standing workstations. However, the sample size of the study was small and the behaviour changes of employees were very variable preventing conclusions from being drawn [9].

Our study was designed to assess the impact of SW stations on the overall PA, especially when high desks are installed at a walking distance from the usual sitting desks and shared between workers. We postulated that the amount of PA is higher during a six-week SW period in comparison to a normal work period.

## Methods

### Participants

The intervention for the present study was conducted at the headquarters of a retail company in Tokyo, Japan. Thirty-two white-collar workers (22 men and 10 women, aged 44.2  ±  8.6 years) participated in the study. Roles of workers were diverse, for example, sales, general affairs, accounting, etc. According to our sample calculation, 25 participants could yield a power of 0.8 based on a satisfying effect size of 0.5 METs · h/day for the difference in PA between groups as the primary outcome. Thirty-two participants were chosen to deal with potential dropouts. Pregnant women, part-time workers, and workers involved in unusual tasks (short-term projects) were excluded from the study. All participants were informed about the purpose of the study and provided written informed consent as approved by the Ethics Committee at the National Institute of Health and Nutrition in Japan (NIHN).

### Study protocol

**Figure 1:**
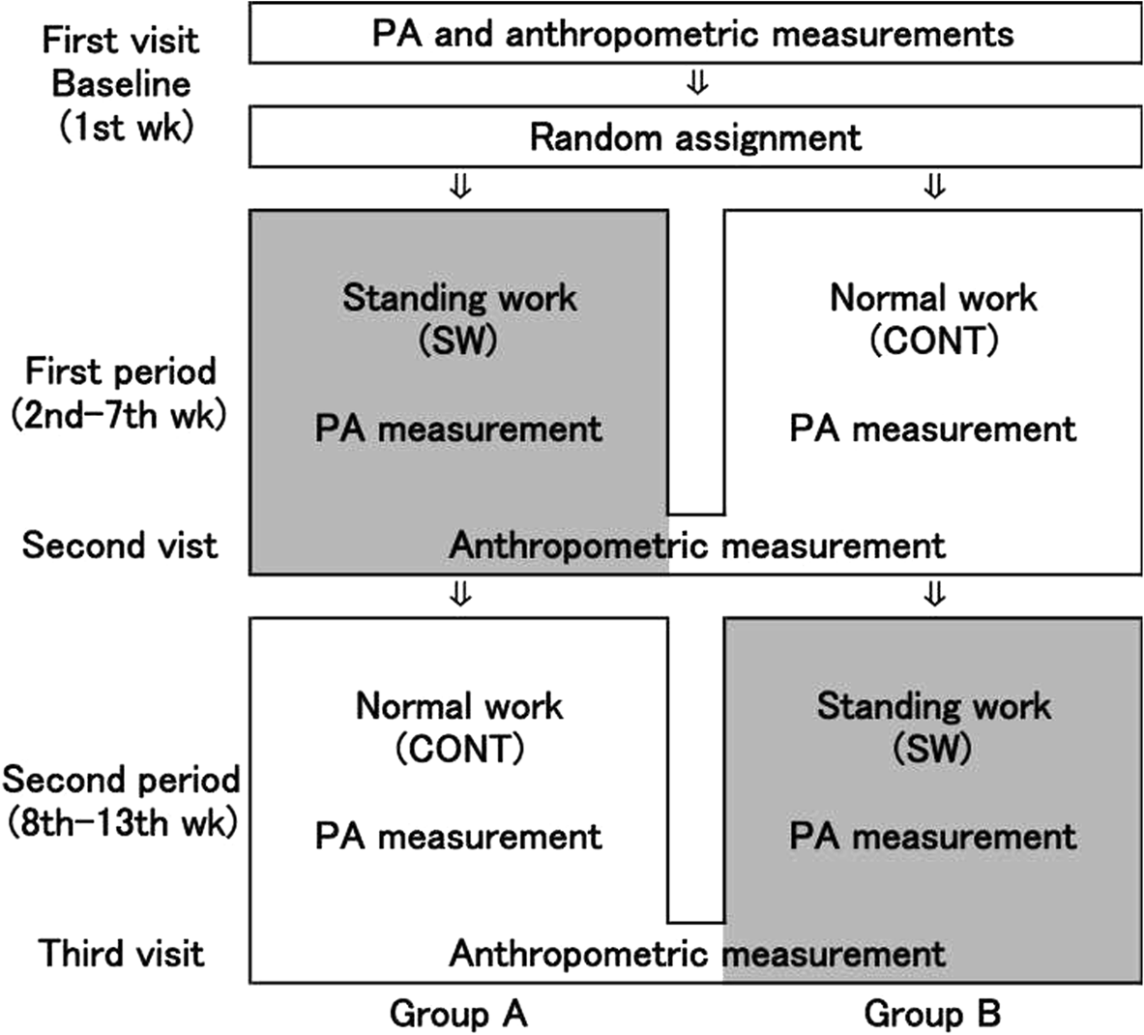
After baseline assessment, 32 participants were randomly divided into two groups. A crossover trial (16 vs. 16) was performed for thirteen weeks including baseline measurements. The participants assigned to Group A completed their office work in a standing position (standing work, SW) for 10 hours per week or more (SW period) during the first experimental period from weeks 2 to 7, and simultaneously the participants of Group B were asked to maintain their normal sitting working habits during the control period (CONT period) after the baseline measurements. At the end of the first period the activity of the group was changed for either CONT (Group A) or SW (Group B) for the second period from weeks 8 to 13. Anthropometric measurements were taken on the 1st week, 7th week, and 13th week, and the comparison of before and after the SW period were analysed. PA assessments were performed throughout 13 weeks, and data extraction from accelerometers was performed at the same time as anthropometric. Statistical comparisons for PA measures were performed between the SW period and CONT period to verify the effects of installation of standing desks.

Figure 1 shows a flow chart of the experimental protocol. Each participant completed a two-phase crossover experimental protocol. During the SW period, subjects were asked to modify their working habits by completing 10 hours of standing work per week. During the control (CONT) period subjects were asked to maintain their normal sitting working habits. Both experimental periods lasted six weeks and were completed successively. The order of the SW and control periods were randomised for each subject. To perform the anthropometric measurements, three review assessments were scheduled: one at the beginning of the protocol (1st week), the second at the end of the first experimental period (7th week) and the third at the end of the second experimental period (13th week). PA was monitored objectively using a waist mounted triaxial accelerometer throughout the thirteen weeks of the study. The 1st week was use to acquire the baseline PA and anthropometric data for randomised assignment as well as helping the participants get accustomed to wearing the device. For the first experimental period, data were collected from the 2nd to the 7th week. For the second experimental period, data were collected from the 8th to the 13th week. To verify an effect of the installation of high desks, the mean PA parameters were compared between SW and CONT periods using a paired *t*-test.

To induce SW, stationary high desks (height: 1000 mm) were specifically installed at the participants’ workplace for the six-week SW period. Participants in the intervention period shared 16 standing desks located approximately 5 – 10 m from their usual sedentary desks in an open-space office. The high desks were removed at the end of the SW period. Each individual participant decided the 10 hour distribution of SW for themselves each week. Subjects were recommended to perform the following tasks during their standing time: writing, reading, PC work and meetings.

### SW record and physical activity measurements

The participants recorded the daily SW times and durations for working days in a diary during the intervention period. The number of people doing 10 hours or more of SW per week was recorded, as well as the mean weekly duration.

The daily amount of PA was measured using a waist-mounted triaxial accelerometer-based PA monitor (Actimarker EW4800; Panasonic Electric Works, Osaka, Japan, cf. references [16] and [17] for validation studies). The PA measurements were performed throughout the 13 weeks, and data extraction from accelerometers was performed at the end of 1st week, 7th week, and 13th week (Figure 1). The daily averages for: 1) the number of steps (cf. step count), 2) the time spent for light PA (LPA, i.e. activities in an intensity range of 1.1 – 2.9 METs), 3) the time spent for moderate PA (MPA, 3.0 – 5.9 METs) and 4) the time spent for vigorous PA (VPA, over 6.0 METs) were recorded. The total PA and moderate-to-vigorous PA (MVPA) are also reported in METs · h/day.

Participants were instructed to wear the accelerometer continuously during their waking hours on both weekdays and weekends. The accelerometer screen was blinded with coloured tape so that the subjects could not have feedback on their current level of PA. For the purpose of accelerometer data processing, a valid day was defined as having at least 10 hours of wear time. Non-wearing days were identified from the participant’s diary record and excluded from the data processing. For each day, the wear time was recorded using the following criteria: from the first to the last acceleration waveforms. Accelerometer-related parameters were analysed in three different ways: weekdays only, weekends only and both weekdays and weekends combined (referred to as “daily”). Only weeks with at least three valid working days and one valid weekend day were included in the study.

### Anthropometric measurements

Height was measured using a stadiometer (YL-65; Yamagi Inc., Nagoya, Japan), body weight was determined using a digital scale (Inner Scan BC-600; Tanita Co., Tokyo, Japan) and BMI was calculated. Waist circumstance was measured at the umbilical level with an inelastic measuring tape at the end of normal expiration. All measurements were performed from 09:00 to 11:00 after an overnight fast. Three assessments were scheduled to take the anthropometric measurements, 1st week, 7th week, and 13th week (Figure 1).

### Statistical analysis

The differences between the two periods for PA outcomes were analysed using a paired *t*-test. For the latter, the analysis was performed for 1) weekdays only, 2) weekends only, and 3) daily. The effect of the installation of high desks was analysed by comparisons of anthropometric parameters before and after the SW period using a paired *t*-test. Statistical analyses were performed using SPSS 20.0 J (SPSS Japan, Inc., Tokyo, Japan). In all analysis, *P*  <  0.05 was taken to indicate statistical significance. Data are presented as means  ±  standard deviation.

## Results

### Baseline characteristics of participants

Baseline characteristics of groups A and B are presented in Table 1. There were no significant differences in anthropometric parameters or PA parameters between groups.

**Table 1:** **Baseline participant’ characteristics**

	**Mean ± SD**	
	**Group A**	**Group B**
N (female)	16	16
Mean age	44.4 ± 6.9	44.0 ± 10.2
Anthropometric		
Height (cm)	169.1 ± 7.0	167.4 ± 8.8
Weight (kg)	66.2 ± 9.6	67.1 ± 14.2
Waist (cm)	82.8 ± 6.3	84.2 ± 9.3
BMI (kg/m2)	23.1 ± 2.5	23.8 ± 3.6
Daily physical activity		
N	16	16
Step counts (counts/day)	9708 ± 1921	9115 ± 2162
Total PA (METs • h/day)	10.3 ± 1.4	10.1 ± 3.7
Time spent of total PA (min/day)	551.2 ± 78.6	513.4 ± 143.8
MVPA (METs • h/day)	3.9 ± 1.6	3.4 ± 1.4
Time spent of MVPA (min/day)	60.1 ± 20.7	53.9 ± 16.8
Time spent of light PA (min/day)	491.1 ± 80.9	459.5 ± 135.3
Time spent of moderate PA (min/day)	57.0 ± 19.0	52.2 ± 15.1
Time spent of vigorous PA (min/day)	3.1 ± 8.4	1.8 ± 4.2
Wearing time (min/day)	962.5 ± 81.0	907.6 ± 89.8

Data are presented as mean ± standard deviation. Total PA, total amount of physical activity; MVPA, moderate to vigorous physical activity; METs, metabolic equivalents.

### Standing work and physical activity metrics

There were no differences in the number of participants completing 10 hours or more of SW per week between groups A (73%  ±  22%) and B (67%  ±  35%). The SW duration was similar in the two groups (9.9  ±  0.9 and 9.6 ± 1.7 hrs/week, respectively). No differences were observed between the two groups for the accelerometer wear time at baseline (Table 1) or throughout the experimental period (data not shown).

The comparisons in PA parameters between SW and CONT periods are shown in Table 2. For the daily and weekdays only, total PA (METs•h/day), MVPA (METs•h/day), time spent in moderate PA (min/day) and time spent in MVPA (min/day) presented significant higher values during the SW periods than during the CONT periods. No significant differences were observed for step counts, time spent in light PA, time spent in vigorous PA, and total PA (min/day).

**Table 2:** **Comparisons in mean PA measures between SW and CONT period**

	**SW period (6 weeks)**	**CONT period (6 weeks)**	***P*** **values**
Daily PA			
N	31	31	
Step counts (counts/day)	10212 ± 2777	9781 ± 2806	NS
Total PA (METs • h/day)	10.2 ± 2.4	9.7 ± 2.3	0.043
Time spent of total PA (min/day)	544.6 ± 117.5	536.1 ± 117.0	NS
MVPA (METs • h/day)	4.2 ± 2.2	3.7 ± 1.8	0.025
Time spent of MVPA (min/day)	62.8 ± 25.1	57.0 ± 20.3	0.019
Time spent of light PA (min/day)	481.9 ± 116.9	479.1 ± 113.5	NS
Time spent of moderate PA (min/day)	58.2 ± 20.7	53.4 ± 17.0	0.019
Time spent of vigorous PA (min/day)	4.6 ± 11.1	3.6 ± 11.6	NS
Weekdays PA			
N	31	31	
Step counts (counts/day)	10714 ± 2588	10254 ± 2782	NS
Total PA (METs • h/day)	10.2 ± 2.5	9.8 ± 2.4	0.047
Time spent of total PA (min/day)	566.2 ± 131.0	555.0 ± 130.8	NS
MVPA (METs • h/day)	4.3 ± 2.0	3.8 ± 1.6	0.035
Time spent of MVPA (min/day)	65.4 ± 22.7	59.9 ± 19.2	0.022
Time spent of light PA (min/day)	500.8 ± 127.7	495.1 ± 125.4	NS
Time spent of moderate PA (min/day)	61.7 ± 19.4	57.3 ± 17.1	0.013
Time spent of vigorous PA (min/day)	3.7 ± 10.8	2.6 ± 10.7	NS
Weekends PA			
N	31	31	
Step counts (counts/day)	9098 ± 4002	8322 ± 3681	NS
Total PA (METs • h/day)	9.9 ± 3.3	9.2 ± 3.1	NS
Time spent of total PA (min/day)	487.5 ± 132.9	467.9 ± 142.3	NS
MVPA (METs • h/day)	4.0 ± 3.3	3.5 ± 2.8	NS
Time spent of MVPA (min/day)	57.1 ± 34.5	50.0 ± 29.6	NS
Time spent of light PA (min/day)	430.4 ± 138.1	417.9 ± 143.8	NS
Time spent of moderate PA (min/day)	51.0 ± 28.3	44.1 ± 23.3	NS
Time spent of vigorous PA (min/day)	6.1 ± 15.7	5.9 ± 15.7	NS

Data are presented as means ± standard deviation. Total PA, total amount of physical activity; MVPA, moderate to vigorous physical activity; METs, metabolic equivalents; NS, not significant.

### Anthropometric measurements

Waist circumference significantly decreased after the SW period (before: 83.7 ±  7.9 cm, after: 83.0 ±  7.9 cm, *p* = 0.007). No other significant changes were noted in other anthropometric outcomes.

## Discussion

To our knowledge, this is the second randomised crossover trial to examine the effects of SW intervention on objectively measured PA [15] and the first using a validated waist mounted accelerometer monitor. We examined how PA was affected by SW intervention over a six-week period. The percentage of participants completing 10 hours or more of SW per week (i.e., meeting the intervention requirement) was approximately 70%. The daily total PA, MVPA and time spent of moderate PA increased significantly during the SW intervention period compared to the CONT period. With weekdays being identified as the main contributor to these observed increases. Waist circumference was also significantly reduced during the SW period. However, no significant changes were observed for the other anthropometric parameters.

### Adherence to the standing work intervention

Our findings suggest that the installation of a standing desk at the workplace may indeed be an effective strategy to reduce sedentary behaviour. This observation is in accordance with the literature [7-12], especially the results of Dutta *et al.* [15] who recently showed a reduction of sitting time in employees undergoing a similar standing work intervention. In the latter study, employees increased their “sense of well-being” without decreasing their productivity.

### Impact of a standing work intervention

Interestingly, our study shows that the reduction of sitting time was associated with an increase in daily PA (including MVPA and total PA). These results were consistent with those of Gardiner *et al.* [18] who showed that an intervention aimed at reducing the sedentary time in older adults also increased the time spent spontaneously in LPA and MVPA. In contrast, our results were inconsistent with those of Dutta *et al.* [15] who did not report such an association. One reason for the inconsistency may be that the participants in the Dutta *et al.* [15] study compensated for higher amounts of activity during workdays by being less active during non-workdays. The protocol used by Dutta *et al.* [15] also aimed to replace 50% of the sitting time by standing (~4 hours/day), and such a strong intervention may have induced physical or mental fatigue [19], which might have dissuaded participants from engaging in other forms of PA, especially weekend activity. Moreover, the equipment used differed between the latter study and our study. Dutta *et al.* [15] used sit-stand desks that could be switched from a sitting to a standing position by pushing a lever, while our study involved the use of two desks for each subject: one traditional sitting desk and one high desk workstation. The installation of the high desk was approximately 5 – 10 m from the usual working desk, which may have induced movements between the two workstations, subsequently increasing MVPA on workdays.

The differences between studies may also have been due to the characteristics of each population, with Japanese workers having different responses to such interventions in comparison with their American counterparts. Changes in consciousness and self-efficacy regarding PA may have participated in the overall increase in PA as suggested elsewhere [20], and cultural and environmental specificities may have impacted these changes in different ways. In addition and similarly to the one-week intervention study presented by Gilson *et al.* [9], the large standard deviation might reflect an important inter-subject variability in our study as well. While our six-week intervention data showed a significant increase in PA, inter-individual variation in the response to SW interventions will have to be considered by employers that would like to used SW as a health promotion tool among their workforce. Taken together, these observations suggest that the interaction between the standing working posture, the sedentary time breaks, the promotion of discrete moments of activity and the related increase in PA maybe responsible for the previously described health benefits [10,12,13]. Additional standing work intervention studies are therefore required to determine the impact of the chosen equipment on sitting behaviour and PA changes and to define the optimal type of intervention depending on the population.

Waist circumference, as a surrogate marker of central adiposity, significantly decreased during the SW period. However, it is still unclear whether this positive change was induced by a reduced sitting time or the increase in total PA and MVPA. On the other hand, the relative short intervention period (six weeks) did not allow us to observe significant changes in other body composition parameters. We postulated that a longer intervention period would generate a subsequent greater accumulation of energy expenditure, which could be able to induce positive changes in body weight and BMI. Longer-term SW studies are required to test this hypothesis.

### Limitations

The present study had several limitations. First, it was difficult to control the content and amount of daily work of each participant during the study, which may have influenced the results. However, the crossover design would have negated this protocol limitation. Second, the accelerometer used in the present study is unable to objectively assess the SW durations and number of breaks in sedentary time. Subjects were asked to report SW durations in a diary record, but future studies should use inclinometer-based monitors as recommended elsewhere [18] to obtain more objective data. The third limitation is external factors. The PA behaviour might also have been affected by a variety of external factors (e.g. related to the workload and role of job, etc.). However, regardless of this limitation, the study suggests that the SW intervention can help workers in different working conditions to be more active.

## Conclusions

A randomised crossover study was performed to clarify the effects of SW on objectively measured PA. Our results indicated that SW, via the installation of high desks in the workplace, increases the daily amount of PA, especially on weekdays. We also show that SW increases PA across different working conditions and that the SW intervention also resulted in a significant decrease in waist circumference. These data suggest SW could be used has a tool by employers to increase PA in the workplace.
